# Hypermethylation of CpG islands in the mouse asparagine synthetase gene: relationship to asparaginase sensitivity in lymphoma cells. Partial methylation in normal cells

**DOI:** 10.1054/bjoc.2001.2000

**Published:** 2001-09

**Authors:** H Peng, N Shen, L Qian, X-L Sun, P Koduru, L O Goodwin, J-P Issa, J D Broome

**Affiliations:** Department of Pathology, North Shore University Hospital, Manhasset, NY 11030; North Shore–Long Island Jewish Research Institute, NY 11030; Department of Leukemia, MD Anderson Cancer Center, Houston, TX 77030, USA; Department of Pathology, New York University School of Medicine, New York, NY 10016

**Keywords:** asparagine synthetase, hypermethylation, mouse, lymphoma, ageing

## Abstract

We have sequenced the promoter region of the murine asparagine synthetase gene and examined its methylation profile in the CpG islands of L-asparaginase-sensitive 6C3HED cells (asparagine auxotrophs) and resistant variants (prototrophs). In the former, complete methylation of the CpG island is correlated with failure of expression of mRNA: cells of the latter possess both methylated and unmethylated alleles, as do cells of the intrinsically asparagine-independent lines L1210 and EL4. A similar phenomenon was seen in normal splenic cells of adult mice. This was age related: no methylation was found in weanlings, but up to 45% of gene copies in animals 18 weeks or older were methylated. It was also tissue related, with methylation occurring rarely in liver cells. The relationship of these changes to oncogenesis is considered. http://www.bjcancer.com © 2001 Cancer Research Campaignhttp://www.bjcancer.com


*British Journal of Cancer* (2001) **85**(6), 930–935
© 2001 Cancer Research Campaign
doi: 10.1054/ bjoc.2001.2000, available online at http://www.idealibrary.com on IDEAL

http://www.bjcancer.com

### Hypermethylation of CpG islands in the mouse asparagine synthetase gene: relationship to asparaginase sensitivity in lymphoma cells. Partial methylation in normal cells

**H Peng[1], N Shen[1], L Qian[1], X-L Sun[1], P Koduru[1], LO Goodwin[2], J-P Issa[3] and JD Broome[1,4]**

[1]Department of Pathology, North Shore University Hospital, Manhasset, NY 11030 and the Department of Pathology, New York University School of Medicine, New York, NY 10016; [2]North Shore–Long Island Jewish Research Institute, NY 11030; [3]Department of Leukemia, MD Anderson Cancer Center, Houston TX 77030, USA

**Summary** We have sequenced the promoter region of the murine asparagine synthetase gene and examined its methylation profile in the CpG islands of L-asparaginase-sensitive 6C3HED cells (asparagine auxotrophs) and resistant variants (prototrophs). In the former, complete methylation of the CpG island is correlated with failure of expression of mRNA: cells of the latter possess both methylated and unmethylated alleles, as do cells of the intrinsically asparagine-independent lines L1210 and EL4. A similar phenomenon was seen in normal splenic cells of adult mice. This was age related: no methylation was found in weanlings, but up to 45% of gene copies in animals 18 weeks or older were methylated. It was also tissue related, with methylation occurring rarely in liver cells. The relationship of these changes to oncogenesis is considered. © 2001 Cancer Research Campaign http://www.bjcancer.com

**Keywords**: asparagine synthetase, hypermethylation, mouse, lymphoma, ageing

Asparagine synthetase (ASNS) is a housekeeping gene responsible for the biosynthesis of L-asparagine (ASN). The gene is expressed constitutively in most mammalian cells and regulates its level of activity in response to the concentration of ASN in the plasma or medium, and the extent of tRNA aminoacylation (Andrulis et al, 1979). In contrast to normal cells, the cells of a number of animal malignancies, especially lymphoid, are ASN auxotrophs that fail to express ASNS. In these, deprivation of exogenous ASN by L-asparaginase (Asnase) induces cell death (Broome, 1963; Boyse et al, 1967). This forms the basis for the clinical use of the enzyme as a therapeutic agent for acute lymphocytic leukaemia (Tallal et al, 1970). In the prototypic mouse system, 6C3HED, an Asnase-sensitive lymphoma cell line originating in the C3H strain (here designated 6C3HED-Ade), lacked ASNS activity, as did various similar lymphoma and other cell lines (Patterson and Orr, 1967; Broome, 1968; Horowitz et al, 1968). However, ASNS activity was present in Asnase-resistant variants of 6C3HED (6C3HED-Ind), which grew out during culture in the absence of ASN or after treatment with subcurative doses of enzyme in vivo. The mechanism responsible for the lack of ASNS in the Asnase-sensitive lymphoma cells as opposed to the Asnase-resistant variants has not been studied in recent years, but in addition to theoretical interest, it may be of clinical significance in understanding the responsiveness of acute lymphocytic leukemia to enzyme treatment both initially and during relapse.

*Received 17 November 2000*
*Revised 14 May 2001*
*Accepted 17 May 2001*

*Correspondence to:* JD Broome

The regulation of ASNS mRNA by amino acid concentration has transcriptional and post-transcriptional components involving both *cis*- and *trans*-acting factors (Guerrini et al, 1993). Within the human ASNS promoter, the amino acid response element is important for both basal activity and regulation of ASNS expression under amino acid starvation. Evidence that indirectly suggested that methylation might play a role in regulating ASNS gene expression came from studies made in conjunction with cloning the human gene. It was shown that transfection with plasmids containing human ASNS cDNA into cells of the ASN-dependent Jensen rat sarcoma was capable of conferring protrophy (Andrulis et al, 1987). However, several transfectants with numerous copies of the cDNA exhibited only basal levels of enzyme activity. Treatment of these transfectant cell lines with 5-azacytidine, an inhibitor of DNA methylation, greatly increased the expression of ASNS mRNA, protein and enzyme activity. This was consistent with the finding that 5-azacytidine caused a marked increase in the number of ASN protrophs in cultures of Jensen cells (Sugiyama et al, 1983). However, no full and direct examination of the methylation status of ASNS has been made until now. Our present experiments have characterized the CpG island in the promoter of the mouse ASNS gene and correlated its hypermethylation with inactivation of gene expression in ASN-dependent lymphoma cells. To our surprise, we found that partial methylation in CpG islands of ASNS also occurred in prototrophs from 6C3HED and in other cell lines that are intrinsically ASN independent. So too, normal splenic cells showed partial methylation, the frequency of which was age dependent. These observations may exemplify a process whose relation to oncogenesis is the subject of much interest (Issa, 1999).

### MATERIALS AND METHODS

### Cell lines and cell culture

6C3HED cells were obtained frozen from DCTDC Tumor Repository (National Cancer Institute, Frederick, MD, USA) and after implantation in C3H/He/Jax mice, tumour was inoculated into Dulbecco's modified Eagles medium (DMEM) with 10% fetal calf serum and ASN supplementation (5 mg/100 ml) and maintained in this medium. ASN-independent 6C3HED cell lines (variants) were obtained from cultures as described (Broome, 1963). These and cells of L1210 and EL4 (American Type Culture Collection) were cultured in the absence of exogenous ASN over periods of 6 months or more before harvesting for further studies.

### RNA preparation and Northern blot analysis

Total cellular RNA was isolated from cultured cells or fresh frozen tissue by Trizol reagent (GIBCO/BRL). Ten μg of total RNA was electrophoresed in 1% agarose gels in the presence of formaldehyde, and blotted onto Nytran membranes by standard capillary transfer and UV cross-linking. All the blots were stained with methylene blue to demonstrate equivalent loading of RNA and to mark the migration of 18S rRNA, 28S rRNA and RNA molecular weight ladders (Bethesda Research Laboratories). A mouse ASNS cDNA probe was prepared by isolating a 1.5 kb fragment from an EcoRI restriction digest of a mouse ASNS total length cDNA clone (Goodwin et al, unpublished data), which was then labelled with $^{32}$P-dCTP using a random primer. After hybridization with this probe, the blots were re-hybridized to the DNA probe of β-actin as internal controls.

### mRNA expression analysis using RT-PCR

RNA was isolated from 6C3HED-Ade and Ind cells as described and from 6C3HED-Ade cells cultured for 24 hours in ASN-containing medium with added 5-aza-2′-deoxycytidine. Complementary DNA was synthesized using reverse transcriptase E (Gibco/BRL) following the manufacturer's instructions. PCR reactions were performed in volumes of 50 μl consisting of 200 mmol/l of dNTPs, 10 pmol primers, 2.5 mmol/l of MgC12, and 2.5 units of AmpliTaq Gold DNA polymerase (Applied Biosystems) with 4 μl of cDNA in the manufacturer's buffer (Applied Biosystems). The primers for amplification of ASNS cDNA were 5′CTCTACCTTTGATGTT 3′ and 5′ GTATTGAAGGGAAACTTC 3′, derived from the cDNA sequence obtained by Goodwin et al (Unpublished data) (Gen Bank Accession #U38940). Mouse GAPDH message was similarly treated as a quantitative control for cellular mRNA. Reactions were carried out in a DNA Thermal Cycler (Perkin-Elmer, Gene Amp 2400) under the following conditions: 94˚C for 10 min, then 94˚C/30 s, 55˚C/30 s, 72˚C/1 min for 35 cycles followed by 7 min at 72˚C. Products were analyzed on a 10% acrylamide gel and stained by ethidium bromide visualized under UV light.

### Isolation and characterization of mouse ASNS promoter

From the cDNA sequence of Goodwin et al (Unpublished data) we expanded the 5′ promoter region by walking upstream of exon one, using Genome Walker™ kits (Clontech, Palo Alto, CA, USA) and a specific primer designed from 5′ end of murine ASNS exon one. PCR products from the libraries produced were cloned into the pGEM®-T Easy vector (Promega, Madison WI, USA) and sequenced using an applied Biosystems PRISM 373 DNA automatic sequencer and a Big Dye terminator cycle sequencing kit. Sequence homology analysis was performed by Blast Search (www4.ncbi.nlm.nih.gov) and CpG island analysis using a programme provided by NIH, Japan (http://www.nih.go.jp/~mhirata/cpg-Per.html).

### DNA isolation and methylation analysis

Genomic DNA was isolated by the standard method of proteinase K digestion and phenol – chloroform extraction. A quantity of 2 μg of genomic DNA was treated with sodium bisulfite to convert all unmethylated cytosines to uracil (Frohmer et al, 1992; Herman et al, 1996) using the protocol from 'Resource on DNA Methylation in Aging and Cancer Web site': (http://www.mdanderson.org/leukemia/methylation). After conversion, DNA methylation analysis was performed by both methylation-specific PCR and bisulfite genomic sequencing. For the latter, primer design and PCR conditions followed the above protocol. Primer set 1 consisted of 5′'TGTTGATGGGATATTGGATTG 3′ and 5′'TCATATAACTAAAACCCAACCC 3′ and primer set 2 consisted of 5′ ATAGAAGTAGAATATTTTTTGG3′, 5′ CCAAAACCAAAAAAAATAAAAC3′. The PCR product was cloned into plasmid PCR 2.1 by the original TA cloning kit (Invitrogen, Carlsbad, CA, USA) and clones from each PCR product were sequenced.

### RESULTS

### ASNS gene expression in 6C3HED cells

In accordance with the lack of ASNS activity, no mRNA of the gene was found in 6C3HED-Ade cells. By contrast, a 2 kb message was expressed in 6C3HED-Ind tumour cells. Messenger RNA of similar size is expressed in normal mouse tissues, particularly in the brain (Figure 1). Under some conditions, tissue

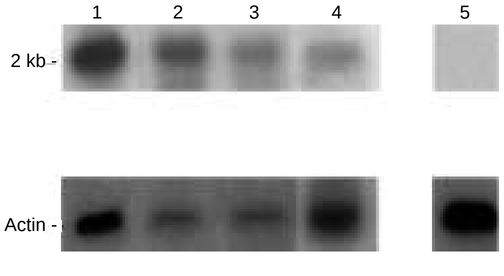

**Figure 1** Northern blot analysis of ASNS in mouse tissues and lymphoma cells. All panels show 10 μg of RNA electrophoresed in 1% agarose gels, blotted, and hybridized with the mouse ASNS cDNA probe (upper panel). The blots were stripped for sequential hybridization with β-actin probe (lower panel). Upper panel: A 2 kb ASNS mRNA was detected in mouse brain (lane 1), spleen (lane 2), kidney (lane 3) and 6C3HED cells in lane 4 (ASN-independent cell line in DMEM with ASN supplementation) after overnight exposure. However, no ASNS mRNA was present in lane 5 (ASN-dependent 6C3HED cells with ASN supplementation in DMEM), even after 1 week's exposure. The lower panel shows the same blot hybridized with β-actin probe

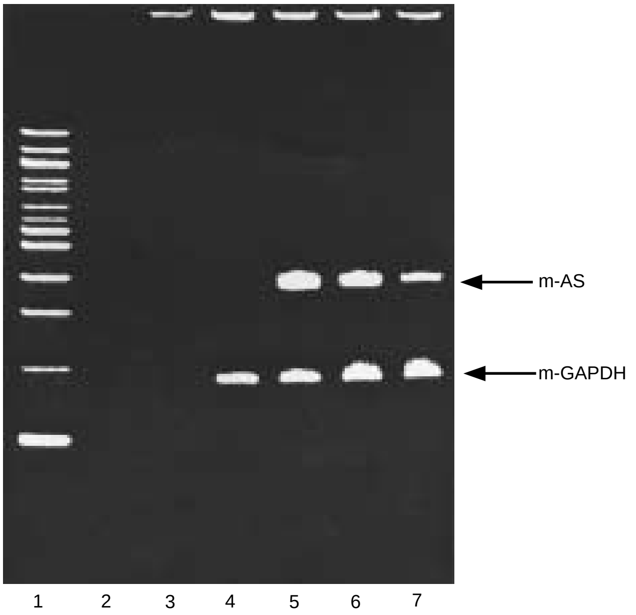

**Figure 2** Effect of 5-AZA-2 deoxycytidine on the transcription of mouse asparagine synthetase in 6C3HED cells. RT-PCR was performed with RNA from 6C3HED-Ade cells (lane 4, negative control), 6C3HED-Ind cells (lane 5, positive control) and 6C3HED-Ade cells cultured with 5 μM of 5-aza-2′ deoxytytidine for 24 h (lane 6) and 10 μM (lane 7). 1 kb molecular marker is shown (lane 1), $H_2O$ control (lane 2), and RNA without reverse transcriptase (lane 3)

cultured cells can also express a 4 kb message, with the additional length being in the 3′ region (data not shown).

To determine whether gene silencing might be due to promoter methylation, we cultured asparagine-dependent lymphoma cells with 5-aza-2′ deoxycytidine, and tested for the expression of the ASNS message by RT-PCR. As shown in Figure 2, distinct expression was induced in 24 h. However, the agent proved toxic and no permanently ASN-independent variants were obtained.

Next we examined directly the methylation status of the promoting region of the gene.

### Isolation and characterization of the promoter of mouse ASNS gene

From 5 Genome Walker libraries, we obtained PCR products from 4. On sequencing, 1 clone showed a high degree of homology with hamster and human ASNS 5′ regions (Greco et al, 1989; Andrulis et al, 1989). The mouse sequence from +1 to –1052 includes the entire untranslated exon 1 and a further 953 bp upstream (GenBank Accession # Bank It 333658 AF 262321). As shown in Figure 3A, starting from –252 to –102, it shares over 80% homology with 5′ regions of hamster ASNS. Interestingly, the short open reading frame (ORF), which precedes the transcription start site, has very high homology with both hamster and human ASNS genes at the DNA and deduced amino acid sequence levels. In the 5′ region, the mouse ASNS gene shares over 70% homology with the human only at this site. Of 14 amino acids of the mouse short ORF, 11 are identical to the human and 12 are identical to hamster (Figure 3A). Strikingly, Guerrini et al (1993) found that mutations in this region not only decreased the basal promoter activity but also abolished amino acid regulation. This highly conserved region is therefore likely to play a major role in the control of ASNS expression. As in both the human and hamster, no TATA box or CCAAT sequences were found in the 5′ region, which is consistent with findings in many housekeeping genes. However, the sequence from –284 to the translation start site is very G + C rich and contains numerous CpG dinucleotides. Sequence analysis confirmed a CpG island in this region (Figure 3B), which therefore had the potential for silencing expression of the ASNS gene by methylation.

### Methylation profile of CpG islands of mouse ASNS gene in lymphoma cell lines and normal mouse cells

To correlate methylation with silencing of ASNS expression in 6C3HED-ADE, we first performed methylation-specific PCR on bisulfite-modified DNA. We found that the DNA of these cells could be amplified by the primer for methylation but not by the primer for unmethylation. But to our surprise, DNA from 6C3HED-Ind was amplified by both types of primer. To analyze the methylation patterns more precisely throughout the whole CpG island of the promoter, we designed 2 sets of PCR sequencing primers for bisulfite-treated DNA. Primer set 1 encompassed –325 to –168 and primer set 2 covered –248 to –37, which together included a total of 22 CpG sites spanning 288 bp. Primer set 1 covered 2 CpG sites upstream, which were not covered by set 2, while the latter conversely included 9 more CpG sites downstream than set 1. Direct PCR product sequencing was performed on DNA from 63HED-Ade, 6C3HED-Ind, and 2 intrinsically ASN-independent lymphoma cell lines: L1210 and EL4. Virtually complete hypermethylation in CpG islands with partial methylation at only a few inconstant CpG sites was found in 6C3HED-Ade but unclear (heterogeneous) sequencing tracings were derived from the other samples. To quantitate the precise proportion of genes with methylated CpG islands, we then cloned the PCR products of bisulfite-treated DNA from the mouse cell lines and sequenced colonies from each PCR-derived clone. We found that all 16 clones from 6C3HED-Ade showed a pattern of virtually complete methylation: 4 clones were methylated in all 22 CpG sites, the remainder had a few (1–4) randomly distributed unmethylated sites. However, surprisingly, 38% (12 out of 32) of clones from 6C3HED-Ind and 44% (4 out of 9) of clones from L1210 were similarly methylated. EL4 showed methylated and unmethylated clones (Table 1). The results demonstrated that the inhibition of ASNS gene expression was only observed with complete hypermethylation of CpG islands. The detailed methylation profile in 19–20 consecutive CpG sites using primer set 2 in 6C3HED cells is shown in Figures 4 A & B. Next we proceeded to examine the status of the gene in normal cells. DNA from weanling mouse spleens showed no methylation of the CpG island. However, in 17-week-old C57 and 18-week-old C3H mice, respectively, 15% (4 out of 27) and 45% (5 out of 11) of the clones were methylated. In an old C3H mouse (48 weeks of age), a high proportion (44%) were methylated. Methylation profiles are shown in Figure 4C&D. The frequency of methylation varied between organs. In the liver, methylation was only seen in one 48-week-old animal, with the very low frequency of 1 clone in 11 (Table 1).

### DISCUSSION

In accordance with much other work (Li, 1999; Ng et al, 1999; Wade et al, 1999) the hypermethylation of the CpGs in the

 © 2001 Cancer Research Campaign

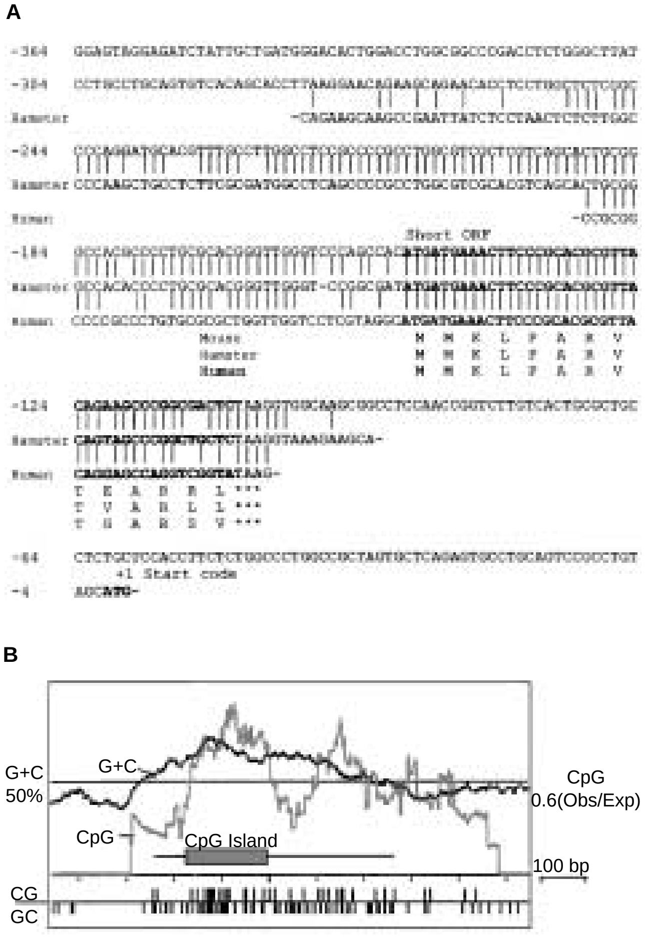

**Figure 3** (**A**) Partial sequence of promoter region of ASNS and demonstration of homology to hamster and human genes. (**B**) Identification of CpG island promoter of ASNS is in all probability the primary cause for its failure to be expressed in ASN-dependent 6C3HED cells, although other secondary changes consequent on methylation are probably involved. In these cells there are two alleles (demonstrated by FISH, data not shown) and hence both must be methylated. On the other hand, most ASN-independent cells probably contain 1 methylated and 1 unmethylated allele. The alternative explanation of a mixed cell population containing either methylated or unmethylated genes is most unlikely. ASN-independent variant lines were grown for at least 50 transfer generations in the absence of exogenous ASN, a condition which would clearly select against dependent cells. Furthermore even after a long period of conditioning (48 hours) by independent cells at high density ($5 \times 10^6$/ml), the medium contains only low levels of ASN (< 10 μM), considerably less than the minimum concentration of 100 μM required to support the growth of dependent cells (Broome, 1963, 1968). Interestingly, we show that in normal mouse spleen, an age-related change appears to be associated with de novo CpG island methylation in the ASNS gene. Furthermore, this methylation is tissue specific. Methylation is high in the spleen but very low in the liver.

In the case of normal spleen cells, since older animals show approximately half the gene copies to be methylated, each cells may contain both a methylated and an unmethylated allele. Alternatively, the degree of methylation may depend on the lymphocytic cell type, with 1 (or more) being unmethylated and the other(s) methylated. The latter would be expected to be Asnase sensitive. Since Asnase is immunosupressive in the mouse (Maral et al, 1979), this possibility merits further study.

The observations in this paper can be considered from a number of more fundamental aspects. First, is the age relationship of methylation in the ASNS gene. It should be pointed out that the differences between weanlings and adults now demonstrated need to be extended to show that the progression of methylation is continuous and to exclude it being solely due to adolescent

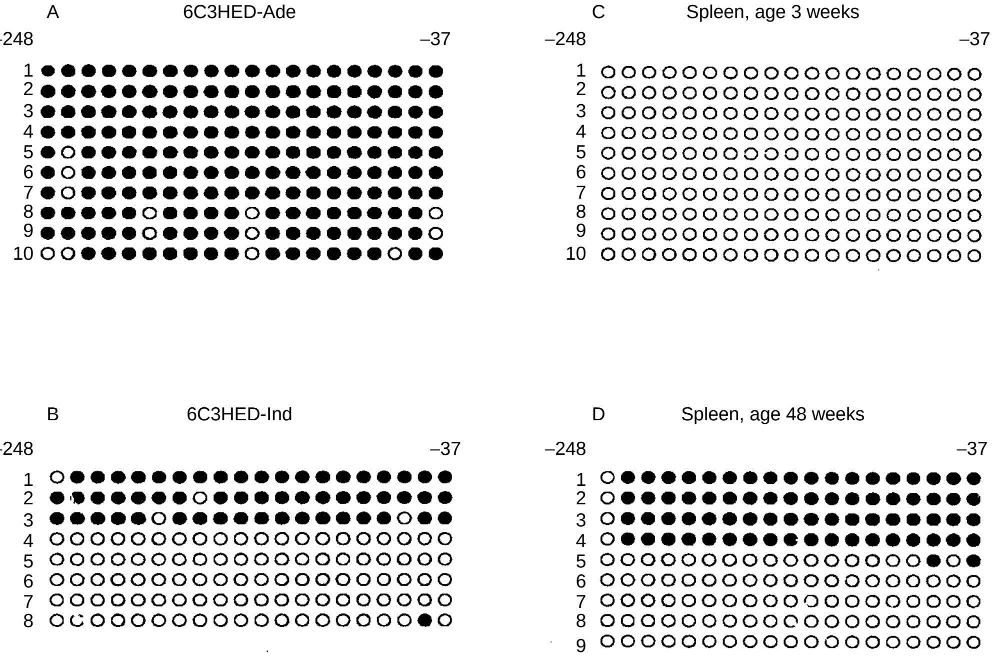

**Figure 4** Methylation profiles of consecutive CpGs in various cell types. Sequencing was performed on products from clones of bisulfite-treated DNA, using primer set 2 (see text). ● Methylated ❍ Unmethylated. Clones are numbered vertically

**Table 1** Methylation of ASNS in DNA from mouse lymphoma and normal cells

| | DNA source | No. of clones sequenced | No. of methylated clones | Methylation proportion (%) |
|---|---|---|---|---|
| Lymphoma cell line | 6C3HED-Ade | 16 | 16 | 100 |
| | 6C3HED-Ind | 32 | 12 | 38 |
| | L1210 | 9 | 4 | 44 |
| | EL4 | 4 | 2 | 50 |
| Spleen (age – weeks) | 3 | 8 | 0 | 0 |
| | 3 | 9 | 0 | 0 |
| | 3 | 9 | 0 | 0 |
| | 3 | 8 | 0 | 0 |
| | 17* | 27 | 4 | 15 |
| | 18 | 11 | 5 | 45 |
| | 48 | 9 | 4 | 44 |
| Liver (age – weeks) | 17* | 9 | 0 | 0 |
| | 18 | 12 | 0 | 0 |
| | 48 | 11 | 1 | 9 |

DNA was obtained from C3H/He, C3D2 F1/Jax or C57 BL/6J (*) mice. The number of methylated clones is the sum of those examined using primer set 1 or primer set 2. Detailed methylation patterns with primer set 2 are shown in Figure 4.

maturation. If this is so, then the methylation falls into a pattern that has been the subject of much interest in various systems (Reviewed by Issa, 1999, 2000). Ageing produces paradoxical effects on methylation in normal tissues. Hypomethylation, primarily of repeated sequences of Alu and satellite DNA is a well-recognized occurrence (Mays-Hoops et al, 1986). By contrast, hypermethylation of CpG islands associated with promoting regions, which in general is linked to silencing of transcription, has been shown to be age associated in an increasing number of identified genes (at least 8) (Issa, 2000). The most comprehensive studies of this phenomenon have been in epithelial cells of the large intestine, prostate and breast, particularly in relation to tumour formation. Until the present study a comparable occurrence in haemopoietic cells has not been reported.

Secondly, genes that have shown age-specific methylation do not necessarily have a gene product that can be associated with cellular growth control, for instance, the muscle-specific MYODI (Rideout et al, 1994). ASNS would appear to be in this category, since there is no prima facie reason that lack of expression of a housekeeping gene would confer a growth advantage. Indeed, the occurrence of methylation may depend on local factors within the chromatin, which are irrespective of function and perhaps relate to changes in methylase activity (Herman and Baylin, 2000). Various mechanisms have been proposed for the spread of methylation from 1 allele to its homolog (Flavel, 1994) but their relevance to the biallelic methylation of ASNS in lymphomas is unknown.

The question may be raised of whether lymphoid cells that show ASNS methylation have an increased propensity to neoplastic change. Both might occur as part of a de novo change in methylation phenotype, the ‘methylome’ (Feinberg, 2001), which amongst other effects can cause the silencing of one or more tumour suppressor genes (Jones and Gonzalgo, 1997; Eng et al, 2000; Costello et al, 2000).

Finally, in whatever way these speculative, but experimentally testable issues may be resolved, the evidence for ASNS inactivation in lymphoid tumours provides a specific example of how a defined epigenetic change can have therapeutic potential.

### ACKNOWLEDGEMENTS

We thank Craig Gawel and Dorothy Guzowski for expert assistance in sequencing and primer synthesis.

### REFERENCES

Andrulis IL, Chen J and Ray PN (1987) Isolation of human cDNAs for asparagine synthetase and expression in Jensen rat sarcoma cells. *Mol Cell Biol* **7**: 2435–2443

Andrulis IL, Hatfield WG and Arfin SM (1979) Asparaginyl t-RNA aminoacylation levels in asparagine synthetase expression in cultured chinese hamster ovary cells. *J Biol Chem* **254**: 10629–10633

Andrulis IL, Shotwell M, Evans-Blockler S, Zalkin H, Siminovitch I and Ray PN (1989) Fine structure analysis of the chinese hamster gene encoding asparagine synthetase. *Gene* **80**: 75–80

Boyse EA, Old LJ, Campbell HA and Mashburn LT (1967) Suppression of murine tumors by L-asparaginase. *J Exp Med* **125**: 17–31

Broome JD (1963) Evidence that the L-asparaginase of guinea pig serum is responsible for its antilymphoma effect. *J Exp Med* **118**: 99–148

Broome JD (1968) Studies on the mechanism of tumor inhibition by L-asparaginase. *J Exp Med* **127**: 1055–1072

Costello JF, Fruhwald MC, Smiraglia DJ, Rush LJ, Robertson GP, Gao X, Wright FA, Feramisco JD, Peltomaki P, Lang JC, Schuller DE, Yu L, Bloomfield CD, Caligiuri MA, Yates A, Nishikawa R, Huang H.-JS, Petrelli NJ, Zhang X, O’Dorisio MS, Held WA, Cavanee WK and Plass C. Aberrant CpG-island methylation has non-random and tumor-type specific patterns *Nat Genet* **25**: 132–138

Eng CE, Herman JG and Baylin SB (2000) A bird’s eye view of global methylation. *Nat Genet* **25**: 101–102

Feinberg AP (2001) Methylation meets genomics. *Nat Genet* **27**: 9–10

Frohmer M, Mc Donald LE, Miller DS, Collis CM, Watt F, Grigg GW, Molloy PL and Paul CL (1992) A genomic sequencing protocol that yields a positive display of 5-methylcytosine residues in individual DNA strands. *Proc Natl Acad Sci USA* **89**: 1827–1831

Greco A, Gong SS, Ittmann M and Basilico C (1989) Organization and expression of the cell cycle gene, ts 11, that encodes asparagine synthetase. *Mol Cell Biol* **9**: 2350–2359

Guerrini L, Gong SS, Mangasarian K and Basilico C (1993) Cis-and transacting elements involved in amino acid regulation of asparagine synthetase gene expression. *Mol Cell Biol* **13**: 3202–3212

Herman JG and Baylin SP (2000) Promoter-region hypermethylation and gene silencing in human cancer. In: Jones PA and Vogt PK (eds), *Current Topics in Microbiology & Immunology* **249**: pp 35–54. Springer-Verlag: Berlin

 *© 2001 Cancer Research Campaign*

Herman JG, Graff JR, Myohanen S, Nelkin BD and Baylin SB (1996) Methylation specific PCR: a novel PCR assay for methylation status of CpG islands. *Proc Natl Acad Sci USA* **93**: 9821–9826

Issa JP (1999) Aging, DNA methylation and cancer. *Crit Rev Oncol Hematol* **30**: 31–43

Issa JP (2000) CpG-Island methylation in aging and cancer. In: Jones PA and Vogt PK (eds), *Current Topic in Microbiology & Immunology* **249**: pp 101–118. Springer-Verlag: Berlin

Jones PA and Gonzalgo ML (1997) Altered DNA methylation and genome instability: A new pathway to cancer *Proc Natl Acad Sci USA* **94**: 2103–2105

Li E (1999) The mojo of methylation. *Nat Genet* **23**: 5–6

Maral R, Guyonnet JC, Julol L, de Ratuld Y and Werner GH (1970) Studies on the immunosuppressive activities of L-asparaginase. *Recent Results Cancer Res* **33**: 160–169

Ng HH, Zhang Y, Hendrich B, Johnson CA, Turner BM, Erdjument-Bromage H, Tempst P, Reinberg D and Bird A (1999) MBD2 is a transcriptional repressor belonging to the MeCP1 histone deacetylase complex. *Nat Genet* **23**: 58–61

Patterson MK and Orr G (1967) L-asparagine biosynthesis by nutritional variants of the Jensen sarcoma. *Biochem Biophys Res Commun* **26**: 228–233

Sugyiana RH, Arfin SM and Harris M (1983) Properties of asparagine synthetase in asparagine-independent variants of Jensen rat sarcoma cells induced by 5-azacytidine. *Mol Cell Biol* **3**: 1937–1942

Tallal L, Tan C, Oettgen H, Wollner N, McCarthy M, Helson L, Burchenal J, Karnofsky D and Murphy ML (1970) E. Coli L-asparaginase in the treatment of leukemia and solid tumors in 131 children. *Cancer* **25**: 306–320

Wade PA, Gegonne A, Jones PL, Ballestar E, Aubry F and Wolff AP (1999) Mi-2 complex couples DNA methylation to chromatin remodelling and histone deacetylation. *Nat Genet* **23**: 62–66

*© 2001 Cancer Research Campaign*
